# Effect of Amaranthus on Advanced Glycation End-Products Induced Cytotoxicity and Proinflammatory Cytokine Gene Expression in SH-SY5Y Cells

**DOI:** 10.3390/molecules200917288

**Published:** 2015-09-18

**Authors:** Warisa Amornrit, Rachana Santiyanont

**Affiliations:** 1 Graduate Program in Clinical Biochemistry and Molecular Medicine, Department of Clinical Chemistry, Faculty of Allied Health Sciences, Chulalongkorn University, Bangkok 10330, Thailand; E-Mail: pollyploycud_36@hotmail.com; 2 Department of Clinical Chemistry, Faculty of Allied Health Sciences, Chulalongkorn University, Bangkok 10330, Thailand

**Keywords:** AGEs, oxidative stress, SH-SY5Y cells, Amaranthus, proinflammatory cytokines

## Abstract

Amaranthus plants, or spinach, are used extensively as a vegetable and are known to possess medicinal properties. Neuroinflammation and oxidative stress play a major role in the pathogenesis of many neurodegenerative diseases, such as Alzheimer’s disease and Parkinson’s disease. Advanced glycation end-products (AGEs) cause cell toxicity in the human neuronal cell line, SH-SY5Y, through an increase in oxidative stress, as shown by reducing cell viability and increasing cell toxicity in a dose-dependent manner. We found that preincubation of SH-SY5Y cells with either petroleum ether, dichloromethane or methanol extracts of *A. lividus* and *A. tricolor* dose-dependently attenuated the neuron toxicity caused by AGEs treatment. Moreover, the results showed that *A. lividus* and *A. tricolor* extracts significantly downregulated the gene expression of the pro-inflammatory cytokines, TNF-α, IL-1 and IL-6 genes in AGEs-induced cells. We concluded that *A. lividus* and *A. tricolor* extracts not only have a neuroprotective effect against AGEs toxicity, but also have anti-inflammatory activity by reducing pro-inflammatory cytokine gene expression. This suggests that Amaranthus may be useful for treating chronic inflammation associated with neurodegenerative disorders.

## 1. Introduction

Oxidative stress and neuroinflammation are common features of chronic neurodegenerative diseases of the central nervous system (CNS), such as Alzheimer’s disease (AD), Parkinson’s disease (PD), Huntington’s disease (HD), amyotrophic lateral sclerosis (ALS), as well as multiple sclerosis (MS). Recent data have described that the CNS is fully immune competent and quickly responds to CNS injury or infections [1]. Alterations of the CNS environment, for example by infection or neuronal injury, result in glia cell activation that can release cytokines and chemokines, as well as reactive oxygen species (ROS). During transient injuries, glia activation leads to the production and release of neurotrophic factors or cytokines that are usually not detrimental to the CNS. However, the prolonged neuronal damage can result in permanent activation of glia and, thus, in constant release of proinflammatory cytokines, such as interleukin-1 (IL-1), interleukin-6 (IL-6) and tumor necrosis factor (TNF), leading to the recruitment of the immune system and the development of a local inflammatory reaction [2,3].

In spite of the diversity of neurodegenerative diseases, oxidative stress due to excessive production and release of reactive oxygen species (ROS), upon mitochondrial injury and dysfunction, has been proposed to be a general pathological mechanism of major chronic neurodegenerative diseases, including AD, PD and MS [2]. Excessive ROS production causes oxidative stress, which can induce cell damage and promote inflammation [4].

Advanced glycation end-products (AGEs) are formed by the reaction of sugars with amino acid side chains followed by oxidations, dehydrations and rearrangements [5]. AGEs cause oxidative stress and cytotoxicity in neuronal cells [6,7], and AGEs-mediated damage is through ROS. An accumulation of ROS leads to oxidative stress that contributes to protein oxidation, leading to protein modification and dysfunction [8].

An increasing number of studies have reported novel therapeutic interventions for neurodegenerative diseases. Natural compounds and supplemental substances have become an increasingly attractive option to treat neurodegenerative diseases, because there is growing evidence that these nutritional constituents have potential adjunctive therapeutic effects, be it protective or restorative, on various neurodegenerative diseases [9]. Several studies have demonstrated that *Ginkgo biloba* has been used to treat a variety of health disorders for centuries. It exhibits several interesting properties, such as an antioxidant property, that can protect the brain from oxidative damage. Furthermore, *G. biloba* can inhibit neurotoxicity and apoptosis. *Panax ginseng* has neuroprotective effects relevant to neurodegenerative diseases, including antioxidants and anti-neurotoxins, that may be derived from its active ginsenosides [10,11].

**Figure 1:**
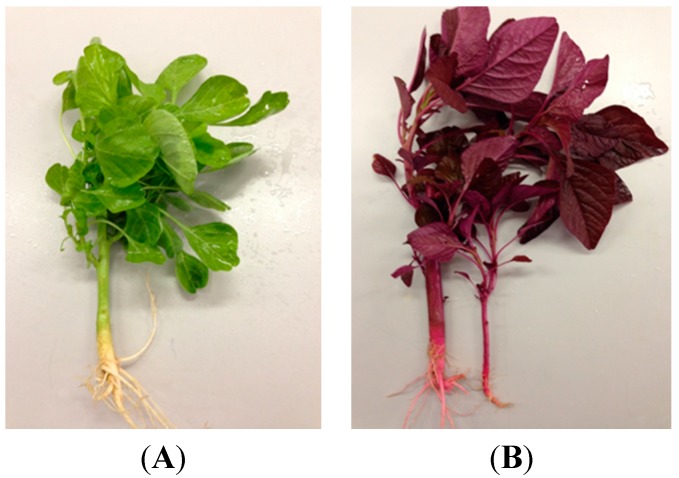
*Amaranthus lividus* L. (**A**) and *Amaranthus tricolor* L. (**B**).

Amaranthus leaves (*Amaranthus lividus* L. and *Amaranthus tricolor* L.; Figure 1) are widely consumed as vegetables in Thailand and are rich in antioxidant components. Amaranthus consists of several antioxidant components, such as polyphenols, flavonoids, betalains, phenolics and anthocyanins [12,13]. Substances containing antioxidants are believed to play a potential role in the treatment of neurodegenerative disorders, such as AD, PD, as well as HD [14,15,16]. The aim of this study was to determine the neuroprotective effect of *A. lividus* L. and *A. tricolor* L. extracts against AGEs-induced cytotoxicity, oxidative stress and proinflammatory cytokine gene expression.

## 2. Results and Discussion

### 2.1. Effect of A. lividus *L.* and A. tricolor *L.* Extracts on Cell Viability in Human Neuroblastoma SH-SY5Y Cells

**Figure 2:**
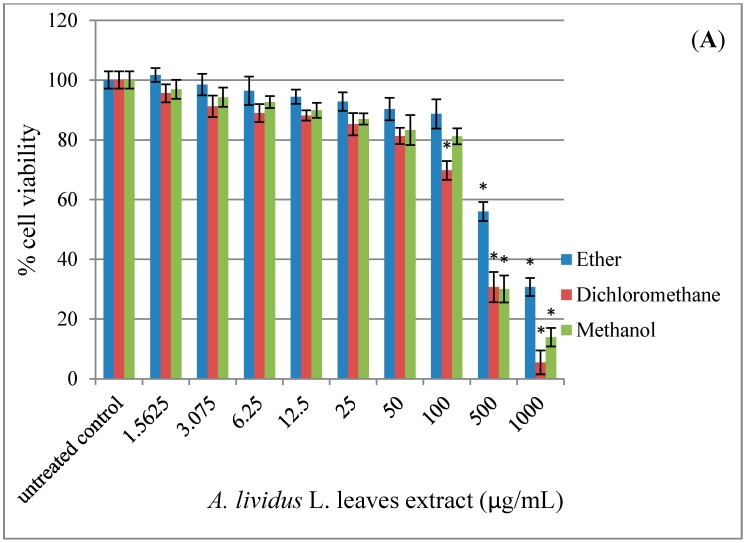

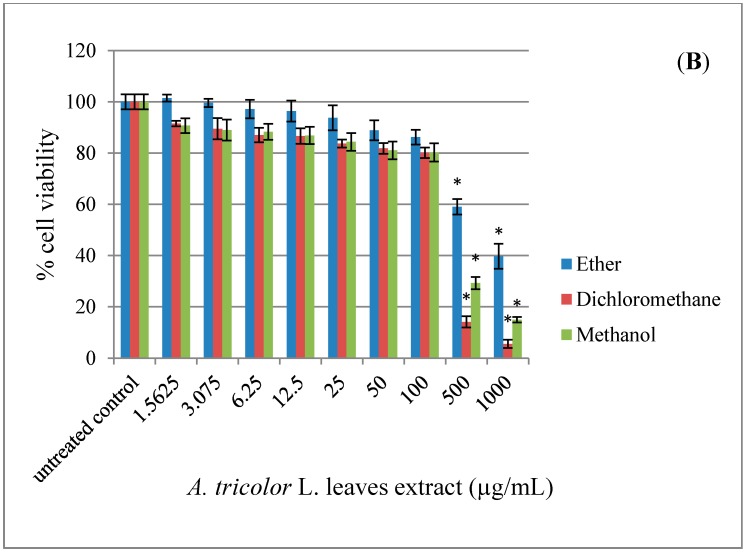
*A. lividus* L. and *A. tricolor* L. extracts have an impact on the cell viability of SH-SY5Y cells. SH-SY5Y cells were incubated with different concentrations of *A. lividus* L. and *A. tricolor* L. extracts (0–1000 μg/mL) for 24 h. The cell viability of living SH-SY5Y cells was assessed using the MTT assay. (**A**) *A. lividus* L. and (**B**) *A. tricolor* L. extracts. Values are reported as the means with their standard error of the mean (SEM), depicted by vertical bars. All experiments were performed in triplicate (N = 3). * *p* < 0.05 for a significant change as compared to untreated control cells.

According to viability test using the MTT assay (Figure 2), exposure of SH-SY5Y cell cultures to *A. lividus* L. and *A. tricolor* L. extracts for 24 h reduced cell viability in a dose-dependent manner (*p* < 0.05). The extracts with petroleum ether, dichloromethane and methanol showed no significant effect on cell viability in the concentration range 1.56–100 µg/mL, except for 1.56–50 µg/mL dichloromethane extract of *A. lividus* L. Cell viability was greater than 80%.

### 2.2. Effect of AGEs on Cytotoxicity in Human Neuroblastoma SH-SY5Y Cells

**Figure 3:**
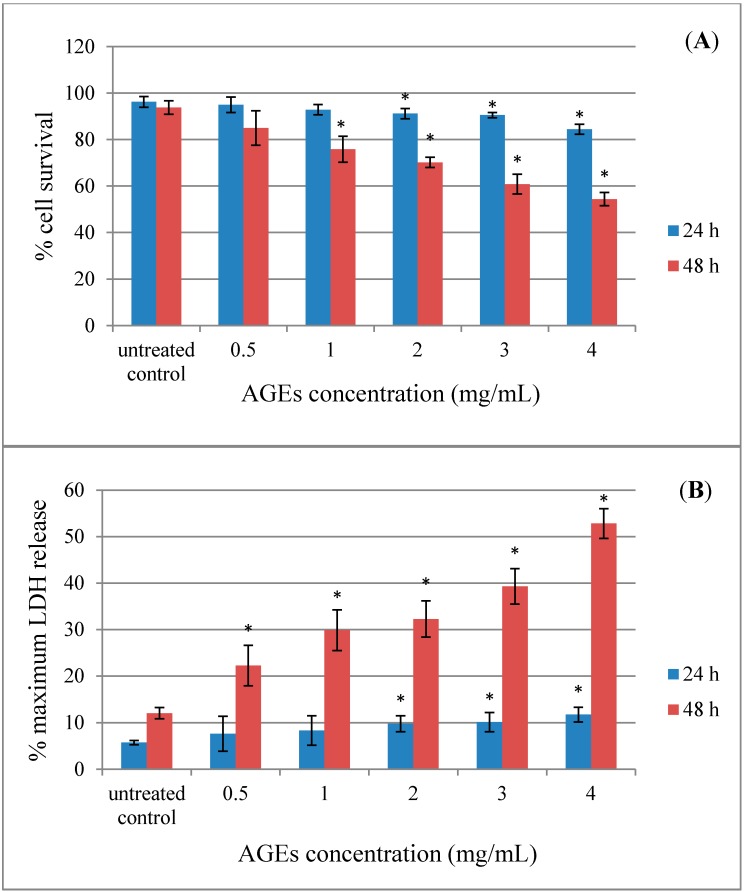
Advanced glycation end-products (AGEs) have an effect on the cell toxicity of SH-SY5Y cells. SH-SY5Y cells were incubated with different concentrations of AGEs (0–4 mg/mL) for 24–48 h. (**A**) The cell survival of living SH-SY5Y cells was assessed using the trypan blue exclusion assay. (**B**) The release of LDH of damaged SH-SY5Y cells was assessed using the LDH assay. Values are reported as the means with their standard error of the mean (SEM), depicted by vertical bars. All experiments were performed in triplicate (N = 3). * *p* < 0.05 for a significant change as compared to untreated control cells.

AGEs are cross-linked structures formed as irreversible byproducts from the cascade of glycation that affect an alteration of the structure and function of tissue proteins [8]. A complex process of protein glycation is initiated by the non-enzymatic interaction between free amino acid groups of protein and the carbonyl group of reducing sugar. The emerging evidence suggests that AGEs can either intramolecularly or intermolecularly cross-link with proteins, leading to protein modification and dysfunction, such as an impairment of enzyme activity, ligand binding and immunogenicity [5]. Glycation-derived free radicals can cause protein fragmentation and oxidation of nucleic acids and lipids [17]. Recent studies have shown that the glycation-associated damage is not limited to patients with diabetes. AGEs have also been implicated in many neurodegenerative diseases, such as HD, ALS and AD [18,19]. Earlier studies indicated that AGEs cause cytotoxicity in neuronal cells [6,7,20]. The extent of the cytotoxicity of AGEs in SH-SY5Y cells was measured using the trypan blue dye exclusion assay (Figure 3A) and the lactate dehydrogenase (LDH) release assay (Figure 3B). Exposure of SH-SY5Y cells to AGEs for 24–48 h reduced cell viability and increased cell toxicity in a dose-dependent manner (*p* < 0.05). The cell morphology of the SH-SY5Y cells changed after exposure to AGEs and detached from the surface. The trypan blue assay showed that cells treated 48 h with 4 mg/mL of AGEs resulted in an approximate 55% reduction in cell viability. The LDH assay measures the release of lactate dehydrogenase from the cell through damage to the cell membrane. Treatment with 4 mg/mL of AGEs for up to 48 h resulted in a 50% increase in LDH above control levels. As the BSA control was produced in a similar manner, but without reducing sugars, a parallel set of BSA treatment was also performed in SH-SY5Y cells. BSA alone showed slight toxicity (data not shown).

### 2.3. Effect of A. lividus *L.* and A. tricolor *L.* Extracts on AGEs-Induced Cytotoxicity

**Figure 4:**
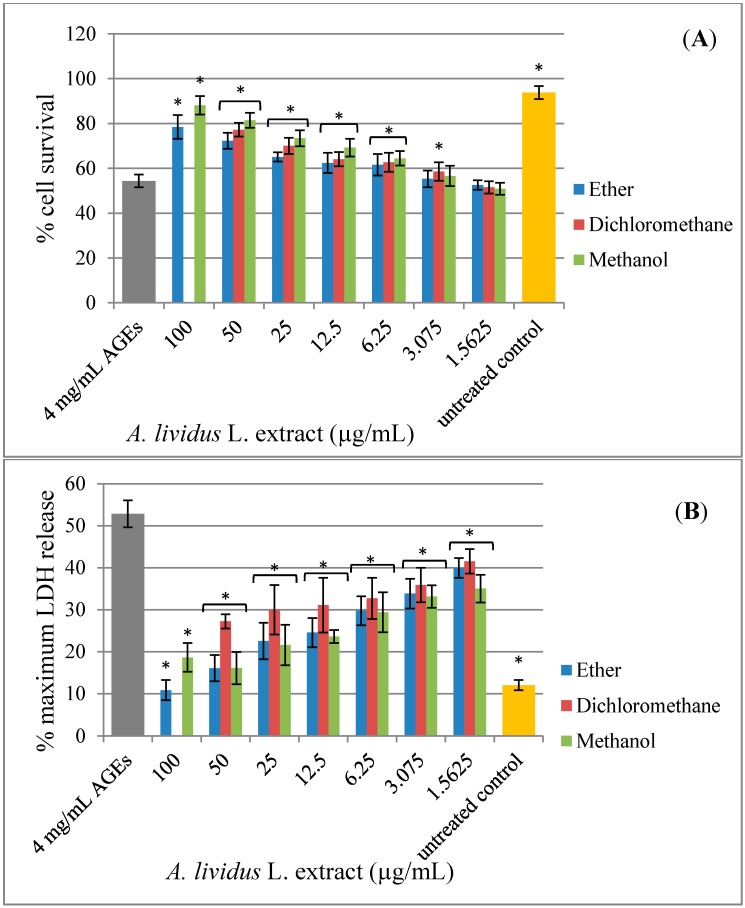
Protective effect of *A. lividus* L. extracts on AGEs-induced cytotoxicity in SH-SY5Y cells. SH-SY5Y cells were preincubated with *A. lividus* L. for 24 h and then further incubated with AGEs for 48 h. (**A**) The cell survival of living SH-SY5Y cells was assessed using the trypan blue exclusion assay. (**B**) The release of LDH from damaged SH-SY5Y cells was assessed using the LDH assay. Values are reported as the means with their standard error of the mean (SEM), depicted by vertical bars. All experiments were performed in triplicate (N = 3). * *p* < 0.05 for a significant change as compared to AGEs treatment alone.

**Figure 5:**
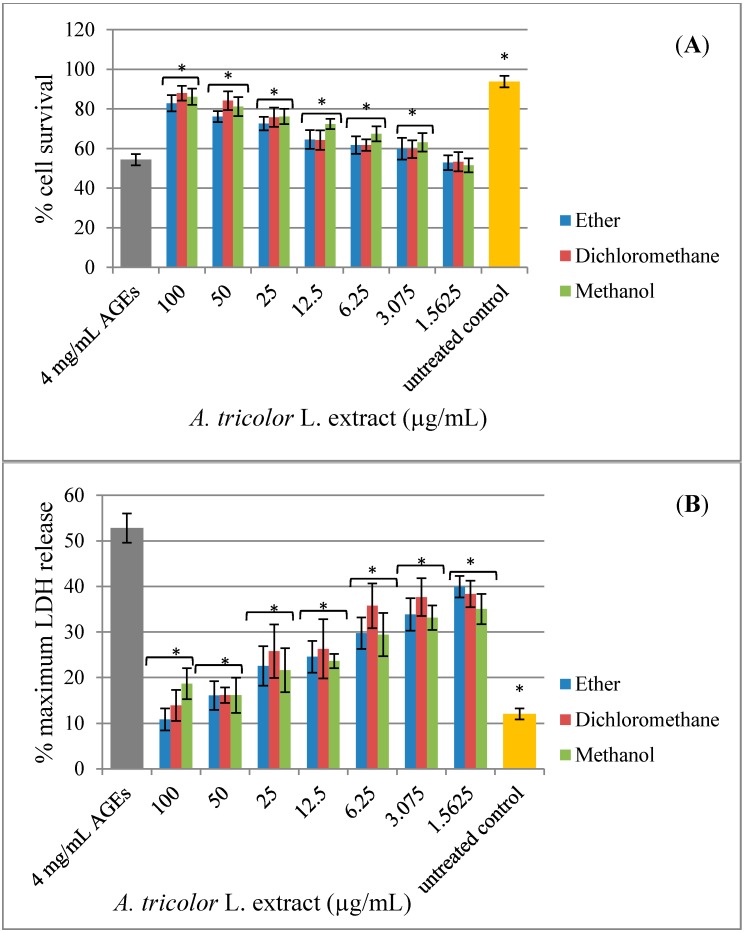
Protective effect of *A. tricolor* L. extracts on AGEs-induced cytotoxicity in SH-SY5Y cells. SH-SY5Y cells were preincubated with *A. tricolor* L. for 24 h and then further incubated with AGEs for 48 h. (**A**) The cell survival of living SH-SY5Y cells was assessed using the trypan blue exclusion assay. (**B**) The release of LDH of damaged SH-SY5Y cells was assessed using the LDH assay. Values are reported as the means with their standard error of the mean (SEM), depicted by vertical bars. All experiments were performed in triplicate (N = 3). * *p* < 0.05 for a significant change as compared to AGEs treatment.

Some studies have shown that Amaranthus extracts contained various types of pharmacologically-active compounds, including antioxidant activity [12,21,22]. Along with the observation that antioxidants, such as N-acetyl-l-cysteine (NAC), vitamin C, vitamin E and carotenoid, can protect cells against AGEs toxicity, these data imply that the cell toxicity is occurring through an oxidative pathway [23,24,25]. The present study aims to investigate the neuroprotective effect of *A. lividus* L. and *A. tricolor* L. extracts against AGEs-induced cytotoxicity in human neuroblastoma SH-SY5Y cells. Exposure of SH-SY5Y cell cultures to AGEs for 48 h significantly induced cell toxicity (Figure 3). *A. lividus* L. extracts with petroleum ether and methanol and *A. tricolor* L. extracts with petroleum ether, dichloromethane and methanol had no significant effect on cell viability; thus, cells can tolerate relatively high doses up to 100 µg/mL; whereas *A. lividus* L. extract with dichloromethane up to 50 µg/mL showed no significant effect on cell viability. When AGEs were applied to the cells pretreated with *A. lividus* L. (Figure 4) and *A. tricolor* L. (Figure 5) extracts for 24 h, the amount of cell toxicity was significantly reduced, suggesting that *A. lividus* L. and *A. tricolor* L. extracts plays a protective role. To measure the extent of the protection against AGEs toxicity provided by *A. lividus* L. and *A. tricolor* L. in SH-SY5Y cells, the trypan blue exclusion assay (Figure 4A and Figure 5A) and the LDH assay (Figure 4B and Figure 5B) were performed. The trypan blue assay showed that cells treated with 4 mg/mL of AGEs resulted in an approximate 55% reduction in cell viability, as shown in Figure 3A. When the cells were pretreated with 3.075–50 µg/mL of *A. lividus* L. dichloromethane extract, 6.25–100 µg/mL of *A. lividus* L. petroleum ether and methanol extracts and 3.075–100 µg/mL of *A. tricolor* L. extracted with petroleum ether, dichloromethane and methanol, there were significant increases in cell viability (*p* < 0.05). In the LDH assay, treatment with AGEs resulted in a 50% increase in LDH above control levels (Figure 3B); whereas pretreatment with of *A. lividus* L. dichloromethane extract (1.56–50 µg/mL), 1.56–100 µg/mL of *A. lividus* L. petroleum ether and methanol extracts and *A. tricolor* L. petroleum ether, dichloromethane and methanol extracts resulted in a significant decrease in LDH release compared to AGEs alone (*p* < 0.05). The present study indicated that *A. lividus* L. and *A. tricolor* L. extracts were capable, in a dose-dependent manner, of attenuating the neuron cell toxicity caused by AGEs treatment. Previous works have shown that compounds with antioxidant activity offer therapeutic potential in the protection against AGEs-mediated cellular toxicity [23]. We suggest that the ability of *A. lividus* L. and *A. tricolor* L. extracts with respect to the neuroprotective effects can likely be associated with their antioxidant properties. Therefore, more investigation into the antioxidant capacity and active compound(s) of extracts from *A. lividus* L. and *A. tricolor* L. is needed to gain more insight into the potential benefit of Amaranthus extracts.

### 2.4. Effect of A. lividus *L.* and A. tricolor *L.* Extracts on AGEs-Induced Generation of Oxidant Stress

**Figure 6:**
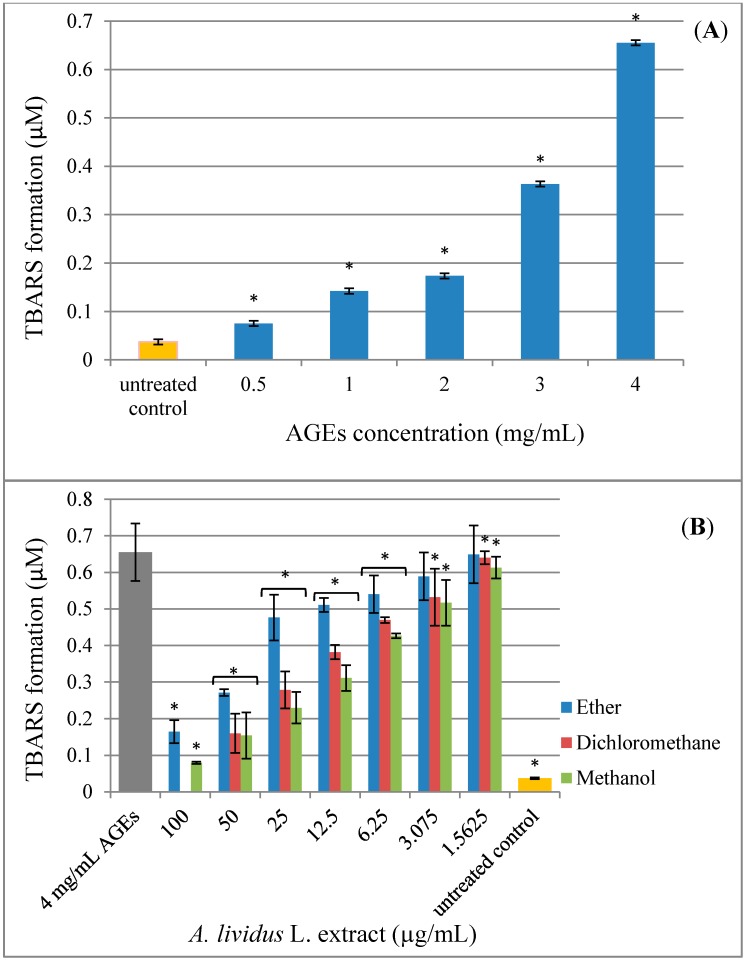

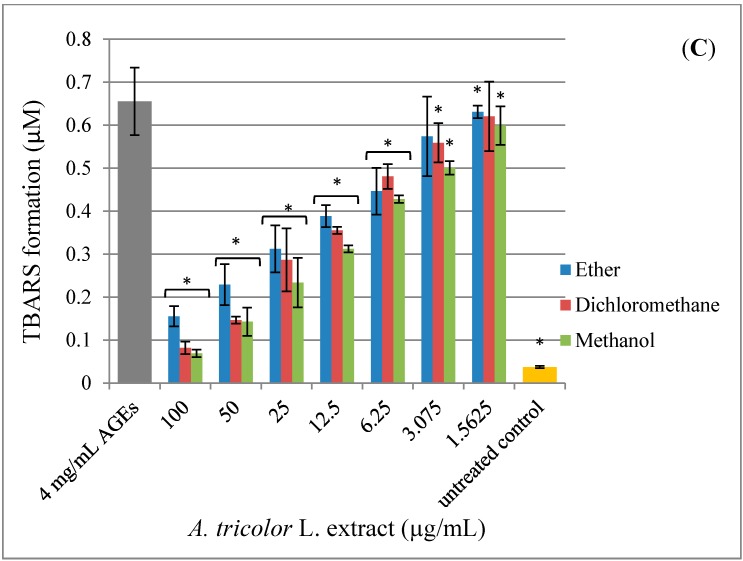
Protective effect of *A. lividus* L. and *A. tricolor* L. extracts on AGEs-induced oxidative stress in SH-SY5Y cells. The generation of thiobarbituric acid reactive substances (TBARS) was assessed using the malondialdehyde (MDA) assay. (**A**) SH-SY5Y cells were incubated with different concentrations of AGEs (0–4 mg/mL) for 24 h. SH-SY5Y cells were preincubated with different concentrations of *A. lividus* L. (**B**) and *A. tricolor* L. (**C**) extracts (0–100 μg/mL) for 24 h. Values are reported as the means with their standard error of the mean (SEM), depicted by vertical bars. All experiments were performed in triplicate (N = 3). * *p* < 0.05 for a significant change as compared to AGEs treatment.

Since the mechanism by which AGEs cause cell toxicity is thought to occur mainly through an increase in oxidative stress [8,26], in order to confirm that the cytotoxicity of AGEs seen in Figure 3 is the result of oxidative stress, thiobarbituric acid reactive substances (TBARS) were used to measure the increase in ROS in the SH-SY5Y cells. ROS cause progressive oxidative degradation of lipids, resulting in the production of a wide variety of oxidation products, including malondialdehyde (MDA), which can be measured as TBARS [27]. The CNS is composed of a high amount of polyunsaturated fatty acids; therefore, oxidative stress can cause damage to cellular membranes and, thus, compromise cell integrity and viability [28]. We used the MDA assay to measure the generation of TBARS during the incubation of AGEs with human neuroblastoma SH-SY5Y cells and to find out whether the *A. lividus* L. and *A. tricolor* L. extracts were capable of reducing oxidative stress in the cells, preventing the toxicity of AGEs (Figure 6). We determined whether exposure of the SH-SY5Y cells to AGEs would lead to the appearance of TBARS in the cells. Generation of TBARS (Figure 6A) occurred when cultured SH-SY5Y cells were incubated with AGEs and was dependent on the AGEs concentration (*p* < 0.05). BSA treatment was ineffective at any concentration (data not shown).

Pretreatment of SH-SY5Y cells with *A. lividus* L. and *A. tricolor* L. extracts also blocked AGEs-induced generation of TBARS, an indicator of oxidative stress (Figure 6B,C). When AGEs were applied to cells pretreated with *A. lividus* L. and *A. tricolor* L. extracts for 24 h, the amount of TBARS formation was significantly reduced, suggesting that *A. lividus* L. and *A. tricolor* L. extracts plays a protective role. The generation of TBARS showed that the cells treated with 4 mg/mL of AGEs resulted in an approximate 0.65 µM TBARS formation. When the cells were pretreated with an *A. lividus* L. concentration of 3.075–100 µg/mL petroleum ether extract, 1.56–50 µg/mL dichloromethane extract, 1.56–100 µg/mL of *A. lividus* L. methanol extract and 1.56–100 µg/mL of *A. tricolor* L. extracted with petroleum ether, dichloromethane and methanol, there was a significant decrease in TBARS formation (*p* < 0.05).

Oxidative stress has been proposed as a general pathological mechanism of major chronic neurodegenerative diseases [2]. AGEs can produce ROS, particularly superoxide and hydrogen peroxide [29,30]. In the body, antioxidant enzymes, such as catalase, superoxide dismutase and glutathione peroxidase, and reducing molecules, such as glutathione, serve as the most potent defense against oxidative stress. However, exogenous antioxidants, such as vitamins A, C and E, also provide protection from oxidative stress [31]. Previous reports identified the presence of the bioactive compounds containing radical scavenging capabilities, such as polyphenols, flavonoids, betalains, phenolics and anthocyanins, in Amaranthus [12,13]. In this study, we found that *A. lividus* L. and *A. tricolor* L. extracts reduced oxidative stress in a dose-dependent manner and attenuated the neuron cell toxicity caused by AGEs treatment. We suggest that the therapeutic effects of the extracts may be due to the combination of various bioactive compounds. Therefore, more studies will be needed to investigate the radical scavenging capabilities in each extract to confirm that the protective effects seen in the present results are from antioxidant activities resulting in cell protection by reducing the toxicity of AGEs by reducing oxidative stress in the cells.

### 2.5. Effect of A. lividus *L.* and A. tricolor *L.* Extracts on AGEs-Induced Expression of Proinflammatory Cytokine Genes in Human Neuroblastoma SH-SY5Y Cells

**Figure 7:**
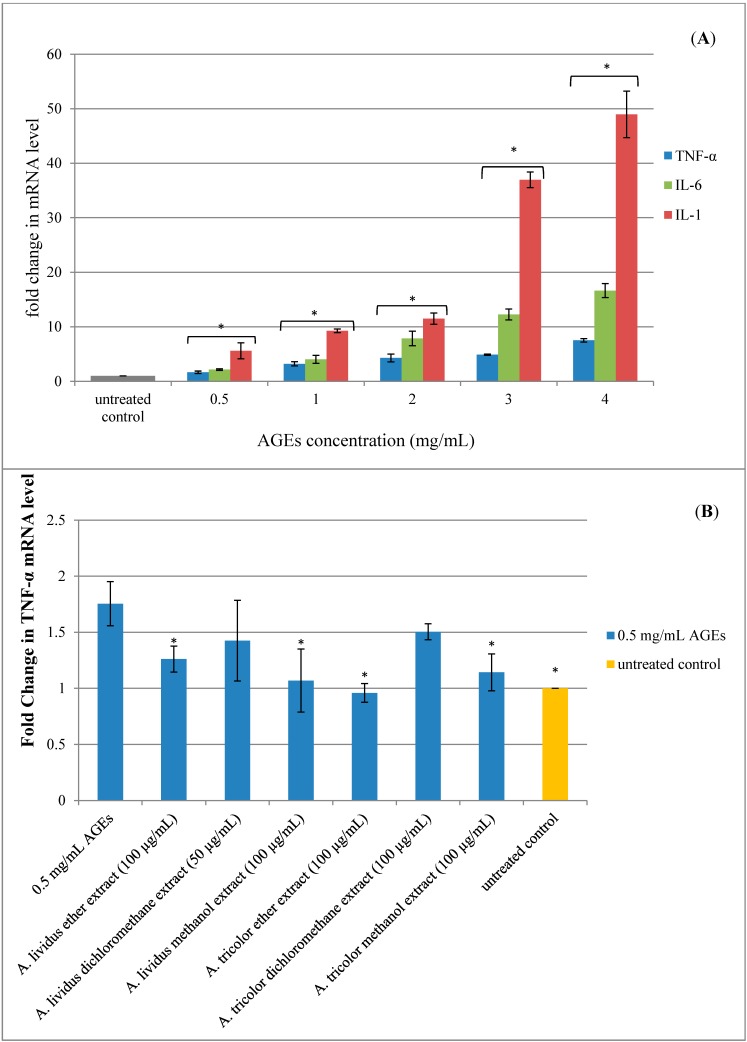

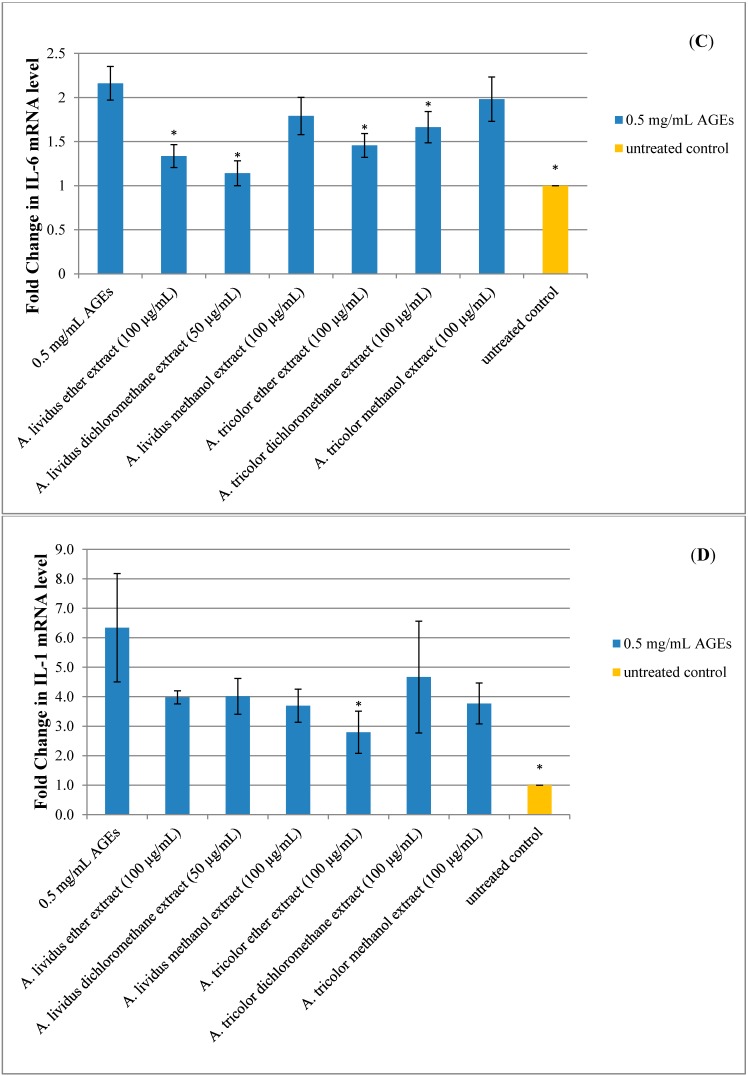
Effect of *A. lividus* L. and *A. tricolor* L. extracts on proinflammatory gene expression in AGEs-stimulated SH-SY5Y cells. (**A**) Human neuroblastoma SH-SY5Y cells treated with different concentrations of AGEs (0–4 mg/mL) for 24 h and TNF-α, IL-6, and IL-1 gene expression levels were determined by qPCR analysis. SH-SY5Y cells were preincubated with *A. lividus* L. extracts and *A. tricolor* L. extracts for 24 h, then further incubated with AGEs for 24 h and qPCR analyses of TNF-α (**B**), IL-6 (**C**) and IL-1 (**D**) were performed. The mRNA expression of GAPDH was used for normalization. Values are reported as the means with their standard error of the mean (SEM), depicted by vertical bars. All experiments were performed in triplicate (N = 3). * *p* < 0.05 for a significant change on the effect of AGEs on gene expression compared to untreated cells, whereas the effect of *A. lividus* L. and *A. tricolor* L. extracts was compared with AGEs-treated cells.

AGEs can produce ROS. Excessive ROS production causes oxidative stress, which can induce cell damage and promote inflammation [4]. Neuroinflammation is an immune reaction to insults, such as stress, injury or infection of the CNS. This inflammatory response in neurons includes activation of microglia, astrocytes, macrophages and lymphocytes, resulting in the release of inflammatory mediators, such as cytokines, chemokines, neurotransmitters and ROS [2]. Currently, the concept has been described that neurons appear to contribute to the production of neuroinflammatory products, while they are traditionally believed to be passive bystanders in neuroinflammation. It has recently been reported that neurons can generate inflammatory molecules, such as IL-1 [32,33], IL-6 [34] and TNF-α [33]. The present study indicated the proinflammatory cytokines gene expression using a quantitative reverse-transcription polymerase chain reaction (qRT-PCR). Exposure of SH-SY5Y cells to AGEs for 24 h significantly induced TNF-α, IL-6 and IL-1 gene expression in a dose-dependent manner, as shown in Figure 7A (*p* < 0.05). However, exposure of SH-SY5Y cells to BSA did not significantly upregulate proinflammatory cytokine gene expression (data not shown).

We found that gene expression in the cells that were incubated with 0.5 mg/mL AGEs significantly increased mRNA expression of TNF-α, IL-6 and IL-1 compared to the untreated control cells, as shown in Figure 7A. Thus, 0.5 mg/mL AGEs were applied to the cells that were preincubated with *A. lividus* L. and *A. tricolor* L. extracts. After preincubation of the SH-SY5Y cells with *A. lividus* L. and *A. tricolor* L. extracts for 24 h, it was found that TNF-α, IL-6 and IL-1 gene expression was significantly decreased (*p* < 0.05) compared to the AGEs-treated cells. TNF-α mRNA expression significantly decreased (*p* < 0.05), as shown in Figure 7B, when preincubated with 100 µg/mL of *A. lividus* L. and *A. tricolor* L. petroleum ether and methanol extracts. Regarding the effect of 100 µg/mL *A. lividus* L. petroleum ether and 50 µg/mL *A. lividus* L. dichloromethane extracts and 100 µg/mL *A. tricolor* L. petroleum ether and dichloromethane extracts, the expression of the IL-6 gene in SH-SY5Y cells (*p* < 0.05) (Figure 7C) was significantly reduced. IL-1 gene expression was significantly decreased (*p* < 0.05) after the cells were incubated with *A. tricolor* L. petroleum ether extract at a concentration of 100 µg/mL for 24 h compared to the AGEs-treated cells (Figure 7D). However, other extracts of *A. lividus* L. and *A. tricolor* L. also indicated a decrease in IL-1 gene expression, but with no significant difference.

This is the first report of the anti-inflammatory effect of *A. lividus* L. and *A. tricolor* L. extracts on AGEs-induced neuroinflammation. Accordingly, due to the constant activation of cells in chronic neurodegenerative diseases, the affected areas are characterized by an increased amount of proinflammatory cytokines, such as IL-1, IL-6 and TNF, which may contribute to disease progression [35]. The present study indicated that AGEs could significantly induce TNF-α, IL-6 and IL-1 gene expression. This result was in agreement with the previous studies that AGEs formation induced cytokines. AGEs from chicken egg albumin could induce TNF-α, and IL-6 production involved both the receptor of advanced glycation (RAGE) and the transcription factor NF-κB [36]. Importantly, the result showed that *A. lividus* L. and *A. tricolor* L. extracts inhibited neuroinflammation by downregulating proinflammatory cytokine gene expression, thereby reduced inflammatory reaction. Previous studies demonstrated that activation of RAGE expressed on the cell surface by binding and stimulation with AGEs leads to intracellular signal transduction involved in aging, inflammation, vascular complications and neurodegeneration. The interaction of AGEs with RAGE causes oxidative stress and activation of nuclear factor-kB (NF-κB) via activation of the p21ras and the mitogen-activated protein (MAP) kinase signaling pathway [37]. NF-kB can regulate gene transcription for the generation of proinflammatory cytokines, such as IL-1, IL-6 and TNF-α [38]. We suggest that the anti-inflammatory effects of *A. lividus* L. and *A. tricolor* L. extracts may be related to the RAGE signaling pathway. Therefore, more studies will be needed to investigate whether the downregulation of the proinflammatory cytokine gene found in this study exerts the effect through the regulation of the RAGE and NF-κB signaling pathways.

## 3. Experimental Section

### 3.1. Plant Material

*A. lividus* L. and *A. tricolor* L. were collected from a single source in Bangkok, Thailand, and identified by Ms. Parinyanoot Klinratana of the Herbarium, Department of Botany, Faculty of Science, Chulalongkorn University, Thailand, Voucher No. 013695 (BCU) for *A. tricolor* L. and No. 013696 (BCU) for *A. lividus* L. The fresh leaves were cleaned with water, dried in a laboratory oven at 45 °C for 5 days and finally ground into a fine powder. The plant powder was successively extracted using a Soxhlet extractor with organic solvents (1:10, *w*/*v*) using series of organic solvents with increasing polarities (petroleum ether, dichloromethane and methanol) until exhaustion. The plant extracts were collected and evaporated using a rotatory evaporator. Crude extracts were dissolved in dimethyl sulfoxide (DMSO) and maintained as 100 mg/mL stock solutions at −20 °C and protected from light. The stocks were diluted with a cell culture medium to get a desirable concentration, and 0.1% DMSO, corresponding to the final concentration of the highest concentration of the extract, was also used to dilute the culture medium.

### 3.2. Cell Culture

The human neuroblastoma cell line SH-SY5Y, a kind gift from Tewarit Sarachana (Faculty of Allied Health Sciences, Chulalongkorn University, Bangkok, Thailand), was used for all experiments. The SH-SY5Y cell line is the neuronal subclone derived from the SK-N-SH human Caucasian cell line. The cells were cultured in a humidified 5% (*v*/*v*) CO_2_-air environment at 37 °C and grown in the MEM/F12 culture medium (Hyclone, Logan, UT, USA) supplemented with 15% fetal bovine serum (FBS) (Hyclone, Logan, UT, USA), 100 U/mL penicillin and 100 μg/mL streptomycin (Hyclone, Logan, UT, USA). Confluent cultures were used, and they were made quiescent when appropriate by a 24-h incubation with medium supplemented with 5% FBS.

### 3.3. Advanced Glycation End-Product Preparation

AGEs-bovine serum albumin (AGEs-BSA) was prepared according to a previously-described method with minor modification [5]. BSA (VWR, Radnor, PA, USA) was modified *in vitro* by the reducing sugar D-glucose (Ajax Fine chem, AUS & NZ) at 37 °C for 12 weeks in the dark. All incubations were carried out in phosphate-buffered saline (PBS; Hyclone, Logan, UT, USA), pH 7.4, and contained sodium azide to prevent bacterial contamination. Control BSA was produced in a similar manner, but without reducing sugars. Unbound sugar was removed by dialysis in PBS. Then, AGEs solution was filtered through sterile syringe filters (0.2 µm) for sterilization and then aliquoted and stored at −80 °C. AGEs were characterized by using a spectrofluorometer with the excitation and emission wavelengths of 355 and 460 nm, respectively. The relative fluorescence of the AGEs was increased approximately 5-fold compared to the non-glycated BSA [39]. Endotoxin levels of AGEs were tested at 1/10 concentrations of the stock solutions for AGEs-BSA; this was below the detection limit of the endotoxin assay (Sigma, St. Louis, MO, USA). Protein content was determined by the Bradford assay, using BSA as the standard.

### 3.4. 3-(4, 5-Ethylthiazol-2-yl)-2, 5-diphenyltetrazolium Bromide (MTT) Assay

Cell viability was determined using the MTT assay, which is the measurement of mitochondrial dehydrogenase enzyme activity that reduces yellow 3-(4, 5-dimethyl-triazolyl-2-yl)-2,5-diphenyl tetrazolium bromide (Bio Basic, Markham, Ontario, Canada) to purple formazan [40]. Briefly, following the cell treatments, SH-SY5Y cells in each well of the 96-well plate were subjected to MTT reagent by mixing with 20 μL of 0.5 mg/mL of MTT solution and incubated at 37 °C in 5% CO_2_ for 4 h. Culture medium was removed, and 200 μL of DMSO were added to dissolve the formazan product; the absorbance was measured at 550 nm using a microplate reader. Since the reduction of MTT occurred in active cells, the level of activity reflected the proportion of viable cells. The percentage of cellular activity was calculated according to the following formula: cellular activity (%) = [(absorbance of treatment group − blank/absorbance of control group − blank)] × 100.

### 3.5. Trypan Blue Exclusion Assay

The trypan blue exclusion assay is used for measuring cell viability. The assay is based on the trypan blue molecule, which is cell membrane impermeable and only enters cells with compromised membranes, thereby rendering the cells a bluish color. This assay allows for a direct identification and enumeration of live (unstained) and dead (blue) cells in a given population. Briefly, following the cell treatments, the cultural medium of SH-SY5Y cells was removed and the cells stained with 0.2% trypan blue (Gibco, Carlsbad, CA, USA) for 3 min. The excess trypan blue was removed and replaced with PBS to prevent the cells from drying out. The cells were then counted under a microscope for 20 high power fields under 40× magnification objective lens; each field did not overlap. The cells were counted by two independent examiners with one who had no prior knowledge of the treatments. The percentage of cell survival was calculated according to the following formula: cell survival (%) = [live unstained cells/(live unstained cells + dead stained cells)] × 100.

### 3.6. Lactate Dehydrogenase Release Assay

Lactate dehydrogenase (LDH) is a stable cytosolic enzyme that is released upon cell lysis. Released LDH in culture supernatants is measured with a 30-minute coupled enzymatic assay, through the oxidation of lactate to pyruvate, which can convert a tetrazolium salt (INT) into a red formazan product. The rate of increase in color formed is directly proportional to the LDH activity of lysed cells [41]. Briefly, following the cell treatments, 50 μL of cultural medium in each well of the 96-well plate were subjected to a fresh microtiter plate; a parallel a set of control and lysed cells was also performed to determine the minimum and maximum release of LDH. Fifty microliters of assay substrate solution (Promega, Madison, WI, USA) were added to this, and the plate was then incubated in the dark for 30 min. Fifty microliters of the stop solution were added, and the plate absorbance was read at 490 nm. The percentage of maximum LDH release was calculated according to the following formula: maximum LDH release (%) = [(absorbance of experimental LDH Release − blank/absorbance of maximum LDH release − blank)] × 100.

### 3.7. Malondialdehyde Assay

This assay is designed to measure thiobarbituric acid reactive substances (TBARS) generated during oxidative stress. ROS cause progressive oxidative degradation of lipids, which results in the formation a wide variety of oxidation products, including malondialdehyde (MDA), which can be measured as TBARS [27]. Measuring TBARS levels offers a method for determining the relative oxidative stress in a sample. Briefly, following the cell treatments, 150 μL of a cultural medium in each well of the 96-well plate were subjected to a fresh microtiter plate, as well as a set of standards (R&D Systems, Minneapolis, MN, USA). Seventy five microliters of thiobarbituric acid (TBA) reagent were added to this, and the plate was then incubated 2–3 h at 45–50 °C; the plate absorbance was read at 532 nm. The generation of TBARS increases in concentration as a response to oxidative stress. The concentration of TBARS in the samples was interpolated from the standard curve.

### 3.8. Quantitative-Reverse-Transcription Polymerase Chain Reaction Analysis

The expression of proinflammatory cytokines genes, including the TNF-α, IL-1 and IL-6 genes, in SH-SY5Y cells was analyzed using the qRT-PCR method. Briefly, total RNA was isolated from SH-SY5Y cells using the TRI reagent (Favorgen Biotech Corp, Ping-Tung, Taiwan) according to the manufacturer’s instructions. RNA was treated with deoxyribonuclease I (DNase I; Promega, Madison, WI, USA). DNase I-treated RNA was reverse transcribed using the ImProm-II Reverse Transcription System (Promega, Madison, WI, USA) following the manufacturer’s protocol. For the amplification reaction, a quantitative polymerase chain reaction (qPCR) was performed in standard 96-well plates using the Exicycler™ 96 Real-Time Quantitative Thermal Block (Bioneer, Daejeon, Korea). The SYBR green system was used to analyze TNF-α, IL-1, IL-6 and GADPH using the following gene-specific primer pairs, as listed in Table 1. SYBR green qPCR was then performed under the following procedures, as listed in Table 2. The fold change in messenger RNA levels was then calculated using the ΔΔct method (2^−ΔΔct^).

**Table 1:** Specific nucleotide primers used in qPCR.

Gene		Sequence	Annealing Temperature (°C)	Product Size (bp)
TNF-α	Forward primer	5ʹ TCTCGAACCCCGAGTGACAA 3ʹ	55	181
	Reverse primer	5ʹ TGAAGAGGACCTGGGAGTAG 3ʹ		
IL-1	Forward primer	5ʹ ACCAAACCTCTTCGAGGCAC 3ʹ	56	300
	Reverse primer	5ʹ CATGGCCACAACAACTGACG 3ʹ		
IL-6	Forward primer	5ʹ GAAGAGAGCCCTCAGGCTGGACTG 3ʹ	64	627
	Reverse primer	5ʹ TGAACTCCTTCTCCACAAGCGC 3ʹ		
GAPDH	Forward primer	5ʹ GAAAGCCTGCCGGTGACTAA 3ʹ	60	370
	Reverse primer	5ʹ TCGCCCCACTTGATTTTGGA 3ʹ		

**Table 2:** qPCR procedure for each gene.

Step	Condition	Cycle (s)
Pre-denaturation	95 °C, 10 min	1
Denaturation	95 °C, 15 s	35–40
Annealing/extension	60 °C, 15 s (GAPDH)	
	55 °C, 15 s (TNF-α)	
	56 °C, 15 s (IL-1)	
	64 °C, 30 s (IL-6)	
Detection	Scan	
Melting		1

### 3.9. Statistical Analysis

All experiments were performed independently at least three times and in duplicate or triplicate. Data are expressed as the mean ± SE of the mean (SEM). Statistically-significant differences were evaluated by one-way analysis of variance (ANOVA) followed by Scheffe’s *post hoc* test using SPSS Version 20.0 for Windows. Differences were considered statistically significant at *p* < 0.05.

## 4. Conclusions

This study demonstrates for the first time that Amaranthus extracts protect neuroblastoma cells against AGEs-induced cytotoxicity. This suggests that Amaranthus may be useful for reducing AGEs-induced oxidative stress and neuroinflammation associated with the risk of brain aging and neurodegenerative diseases. However, additional studies are necessary to elucidate the effect of Amaranthus extracts for the exact mechanisms involved in the AGEs signaling pathway to gain more insight into the potential benefit of Amaranthus extracts.
